# Genotoxicity and acute and subchronic toxicity studies of a bioactive polyoxometalate in Wistar rats

**DOI:** 10.1186/s40360-017-0133-x

**Published:** 2017-04-05

**Authors:** Xiaofeng Qu, Kun Xu, Chao Zhao, Xiuling Song, Jinhua Li, Li Li, Wei Nie, Hao Bao, Juan Wang, Fenglan Niu, Juan Li

**Affiliations:** School of Public Health, Jilin University, Changchun, Jilin China

**Keywords:** Polyoxometalate, Acute oral toxicity, Subchronic oral toxicity, Genotoxicity

## Abstract

**Background:**

Cs_2_K_4_Na [SiW_9_Nb_3_O_40_] (POM93) is a novel broad-spectrum antiviral agent with high activity, high stability, and low toxicity in vitro. Most toxicity studies for POM93 have been performed in cultured cell lines rather than in animals. Like other POMs, there is a lack of evidence for in vivo toxicity limits, oral bioavailability, and therapeutic applications.

**Methods:**

The toxic properties of POM93 were evaluated comprehensively in vivo, including the acute and subchronic oral toxicity studies and genotoxicity tests.

**Results:**

The acute toxicity study showed no abnormal changes or mortality in rats treated with POM93 even at the single high dose of 5000 mg/kg body weight. In the subchronic toxicity study, regardless of the body weight, the organ weight, and the hematological parameters, similar results were observed between the control group and the experimental groups. POM93 produced mild changes in rare hematological parameters in the liver and kidneys, but did not induce the clinical symptoms of liver or kidneys injury in rats as confirmed by histopathological analysis. Moreover, neither mutagenicity nor clastogenicity was caused by POM93 treatment in vitro and in vivo.

**Conclusions:**

The present study demonstrates that the oral administration of POM93 is presumed safe and poses a low risk of potential health risks.

**Electronic supplementary material:**

The online version of this article (doi:10.1186/s40360-017-0133-x) contains supplementary material, which is available to authorized users.

## Background

Polyoxometalates (POMs) are polyanionic oxides of early transition metals with unique physicochemical properties (such as polarity, redox potentials, surface charge distribution, shape, and acidity), and have a broad and versatile range of potential applications in the fields of catalysis [1], electrochemistry [2], materials science [3], etc. During the past three decades, POMs have been reported to exhibit biological activities in the aspects of antitumor [4], antiviral (in particular anti-HIV and anti-HBV), antibacterial [5, 6], and recently, anti-Alzheimer [7]. For example, [NH_3_Pr^i^]_6_[Mo_7_O_24_]•3H_2_O (PM-8) has been observed to show significant antitumor activity toward colon adenocarcinoma (Co-4) human colon cancer, mammary carcinoma (MX-1) human breast cancer, and human small cell carcinoma of lung (OAT) [8, 9]. POMs with a Wells-Dawson structure (POMD) were found to efficiently inhibit amyloid β (Aβ) aggregation by binding to the cationic cluster from His13 to Lys16 (HHQK) [10]. The progress in this field has greatly enriched the knowledge of the development of POMs for medicine and/or biosystem applications, and demonstrated that POMs are important drug candidates. Therefore, evaluation of the in vitro and in vivo toxicity of POMs is desirable for their further application in humans and clinical therapy.

Intriguingly, one of the principle advantages offered by POMs is the possibility to design and modify the structure of metal-oxide cluster anions, whereby many elements could be easily introduced into the POM frameworks [11]. In recent years, for minimizing the toxicity of POMs to normal cells, studies have indicated reduced POM toxicity through discovering of new POMs [12], such as encapsulating POMs in biomaterials (such as zeolites and biodegradable/biocompatible hydrogels) [13, 14], and conjugating POMs with organic ligands [15]. Technically, these strategies improve POMs’ biocompatibility [16], allow selective recognition of biological targets [17], and tune the bioactivity and cytotoxicity of POMs [18, 19]. Unfortunately, using POMs for therapy is still in its infancy; the most current research assesses the toxicity of POMs in vitro [12, 19, 20], rarely POM products have been evaluated for their toxic side effects in vivo. More importantly, investigation of acute and subchronic toxicity is essential before the development of POMs for clinical trials, because of the mechanism and consequence of toxicity in vivo was different from that of in vitro.

In our previous work, we initially utilized a green and eco-friendly approach to synthesis various types of POMs. A series of antiviral and biochemical experiments was performed for screening, and it was found that a Keggin-type of POM with the formula of Cs_2_K_4_Na[SiW_9_Nb_3_O_40_] (POM93) possessed high anti-hepatitis B virus (anti-HBV) [6, 21] and anti-hepatitis C virus (anti-HCV) [22] activities with low cytotoxicity on MDCK, Vero, and MT-4 cells. Limited knowledge is available regarding the effects of POMs on human physiology. Thus, in the present study, we used POM93 as a model substrate to prospectively report the acute and subchronic toxicity in Wistar rats. Moreover, genotoxic effects were evaluated in vitro and in vivo. To the best of our knowledge, the systematic toxicity assessment has sparsely been investigated and certainly needs further extension and detailed study.

## Methods

### Preparation of POM93

Cs_2_K_4_Na [SiW_9_Nb_3_O_40_] was prepared as described in the previous literature [23]. Briefly, 6.5 g of K_7_H [Nb_6_O_19_] was dissolved in 400 mL of deionized (DI) water, and 11.6 M hydrogen peroxide was then added with gentle stirring. After the reaction mixture was acidified with 3 M HCl (20 mL), 25.1 g of A-α-Na_9_H [SiW_9_O_34_] was mixed. The mixture was diluted with an additional 300 mL of DI water until the color of the solution turned yellowish, and 10 g of cesium chloride was then added. After the solution was evaporated at room temperature, the yellow precipitate was filtered and collected. The raw product was further recrystallized using DI water to obtain yellow-colored Cs_2_K_4_Na [SiW_9_Nb_3_O_40_] (the X-ray powder diffraction pattern of POM93 is shown in Additional file 1: Figure S1 in the Supporting Information, SI). Elemental analysis: calculated (found): Cs 8.67% (8.97%), K 5.09% (4.93%), Nb 9.09% (9.19%), W 54.02% (54.15%). FT-IR (KBr, cm^−1^): 3429, 2357, 1623, 990, 958, 868, 789, 673, 595, 534, and 474 (see Additional file 1: Figure S2 in the SI).

### Analysis of antimutagenic effects: ames test [24]

The antimutagenic effects of different POM concentrations (8.0 to 5000 μg/plate) were tested using the *Salmonella typhimurium* histidine auxotrophs strains TA97, TA98, TA100, and TA102 for frameshift and base-pair substitution mutagenesis. 2-Nitrofluorene (2-NF), 2-anthramine (2-AA), and sodium azide phosphate were used as the positive control. Mutagenicity was determined by the incorporation method in the presence and absence of S9 metabolic activation [25]. One hundred microliters of each test bacterial culture (1 × 10^8^ cells), 2 mL soft agar (0.6% agar, 0.5% NaCl, 5 mM histidine, and 50 mM biotin, pH 7.4, 40–50 °C), 0.5 Ml S9 mixture (if necessary), and test compounds (positive control chemicals and POM) were mixed well in a test tube. After preincubation at 37 °C in a shaking water bath for 30 min, 2 ml of top agar containing 10% histidine/biotin solution was added and then spread on a minimal glucose agar plate. Following incubation at 37 °C for 2–3 days, the number of his^+^ revertants were counted and the percent inhibition induced by the extract treatment was calculated.

### Experimental animals

Adult male and female Wistar rats (weighing 200 ± 20 g) and ICR mice (weighing 20 ± 2 g), were purchased from the Laboratory Animal Center of Jilin University, China. The rats were separated by sex, housed in clean stainless steel cages, quarantined, and acclimated for 1 week under a 12:12 h light:dark cycle at an ambient temperature of 24 ± 1 °C, and 55 ± 5% relative humidity. Water and food were available ad libitum. All animal studies were approved by the Animal Care and Use Committee of Jilin University (Permit Number: JLU2007-0003).

### Chromosomal aberration assay [26]

Male ICR mice (*N* = 6) were administered test compounds orally by gavage at doses of 0.625, 1.25, and 2.5 g/kg body weight. Negative controls received an equal volume of vehicle, and positive controls were administered an intraperitoneal injection of 40 mg/kg body weight cyclophosphamide. After 24 h of given dose, the animals were anesthetized with carbon dioxide and bone marrow cells were harvested. Two hours before the harvest of cells, Colchicine was administered i.p. of 4 mg/kg body weight. The slides were prepared originally according to the modified method of Perston et al. (1987) [27]. Briefly, mice femur bones were excised and the bone marrow extracted in 0.9% NaCl. The harvest cells were incubated at 37 °C for 20 min and collected by centrifuge at 1000 rpm for 10 min. The cells were then fixed and washed in a mixture of methanol and acetic acid (3:1, v:v), and burst open on clean glass slides to release chromosome. Slides were stained with 5% Giemsa solution for 15 min, immersed in xylene, and mounted with DPX. A total of 100 well-spread diploid M1 metaphase cells per treatment were classified and scored by light microscopy. Different types of chromosomal aberrations such as chromatid breaks, gaps, centromeric association, etc. were scored and expressed as % chromosomal aberrations. Data are expressed as the total mean number of chromosomal aberrations per treatment ± SD from 100 cells scored in 3 independent experiments.

### Micronucleus assay [25]

According to the acute toxicity test results, the most appropriate dose was selected for the erythrocyte micronucleus formation test. Three groups of 5 ICR mice each were administered test compounds orally by gavage at doses of 0.625, 1.25, and 2.50 g/kg body weight. Negative controls received the vehicle and positive controls (cyclophosphamide) were administered an intraperitoneal injection of 200 mg/kg body weight. Peripheral blood samples were collected by perforating the caudal vein at 36 h after administration of treatments, and drops were smeared on a glass microscope slide, and stained with acridine orange as described by Hayashi and Sofuni, 1994 [28]. The micronucleated cells on the prepared slides were immediately scored by fluorescence microscopy. The ratio (%) of polychromatic erythrocytes (PCEs) to total erythrocytes and the frequency of micronucleated polychromatic erythrocytes (MNPCEs; ‰) were analyzed by counting a total of 1000 peripheral reticulocytes or PCEs in three slides per mouse, respectively.

### Acute toxicity study in rats [29]

Thirty healthy Wistar rats were randomly divided into 3 groups (5 males and 5 females per group). On the day of treatment, food but not water was withheld overnight. Group 1 (control) received sterile water, given orally. Experimental groups (2–3) were orally treated with test compound at single doses of 2500.0 and 5000.0 mg/kg, respectively. Food was withheld for a further 3–4 h after giving the treatment. Animals were observed individually for general behavioral and body weight changes, toxic symptoms, and mortality during the first 30 min, periodically during the first 24 h, and at daily intervals thereafter for a total 14 days. At the end of the study (on day 14), all surviving rats were anesthetized with carbon dioxide and the median acute toxicity values (LD_50_) were estimated [30]. The LD_50_ is greater than 5000 mg/kg if three or more rats survive.

### 13-week subchronic toxicity study in rats [31]

Eighty healthy Wistar rats were randomly divided into 4 groups (15 males and 15 females per group). Group 1 (control) received vehicle daily by oral gavage throughout the course of the study. Experimental groups (2–4) were orally administered test compound at doses of 62.2, 195.7, and 587.0 mg/kg body weight/day, respectively, for 13 weeks. Body weight, the amounts of food consumption, and water intake were recorded weekly. At the end of the study, the physiological condition of the rats was restored for another 2 weeks (with food and water supplied ad libitum). Surviving rats were anesthetized with carbon dioxide and blood samples were obtained from rat eyes using capillary tubes for hematological and serum biochemical studies at 45, 90, and 105 days respectively. After blood collection, the rats were sacrificed by cervical dislocation, and their organs were excised (brain, heart, liver, spleen, lungs, kidneys, uterus (or testis), and ovary (or epididymis), stomach, and gut), weighed using an analytical balance and examined macroscopically. All the organs were then finally fixed in 10% neutral buffered formalin for histopathological examination.

### Histopathology [32]

Histopathological analysis was carried out on the preserved organs and tissues. The organs listed above were harvested and fixed in 10% neutral buffered formalin for 48 h. The fixed organs were processed for paraffin embedding. Paraffin Sections (5 mm thick) were cut using a standard microtome, processed with the alcohol: xylene series, stained with hematoxylin and eosin (H & E) and observed by light microscopy.

### Statistical analysis

Statistical analysis was carried out using SPSS 19.0 statistic software package. All data collected were expressed as mean ± standard deviation (SD) and were analyzed using the one-way analysis of variance (ANOVA). Significant differences between the control and experimental groups were determined by Dunnett multiple comparison tests and the differences were considered significant if *P* < 0.05.

## Results

### POM93 treatment caused neither mutagenicity nor clastogenicity in vitro and in vivo

Mutagenicity of the POM93 was evaluated in a bacterial reverse mutation assay in four Histidine-requiring strains of *S. typhimurium* (TA97, TA98, TA100, and TA102). In the Ames test, a remarkable increase in the number of revertants was observed in all the positive control groups compared to the negative control (*P* < 0.05). After treatment with various concentrations of POM93, none of the test concentrations (5000, 1000, 200, 40, or 8 μg/plate) resulted in a clear growth in the number of colonies with or without S9 metabolic activation in all four test strains (Table 1). Furthermore, there was no cytotoxicity in all bacterial systems used in the mutation assay and no dose-dependent increase of revertant colonies was detected for any of the bacterial strains (Table 1).

**Table 1:** Number of revertant colonies after POM93 exposure in absence and presence of S9 mix

Concentration (μg/plate)	Type of bacteria (mean ± SD)			
	TA97	TA98	TA100	TA102
*A. Without S9 mix*				
5000	139.7 ± 14.5	33.7 ± 5.8	162.7 ± 14.0	264.0 ± 24.4
1000	158.7 ± 29.2	36.3 ± 3.7	187.7 ± 16.6	280.0 ± 23.5
200	168.7 ± 12.0	35.7 ± 6.3	162.0 ± 9.5	290.3 ± 16.0
40	167.7 ± 9.0	41.0 ± 3.6	179.7 ± 4.5	273.7 ± 36.6
8	165.3 ± 21.2	32.7 ± 6.6	160.0 ± 22.1	297.7 ± 10.0
0	150.7 ± 5.0	33.0 ± 3.6	171.3 ± 6.8	288.7 ± 10.0
Positive control	1748.7 ± 39.5*^a^	2118.7 ± 94.0*^b^	1760.0 ± 59.7*^c^	1817.7 ± 24.1*^d^
*B. With S9 mix*				
5000	171.3 ± 11.7	40.7 ± 5.0	167.0 ± 38.0	284.3 ± 13.0
1000	163.3 ± 14.3	41.7 ± 2.5	155.0 ± 32.5	272.0 ± 34.0
200	184.0 ± 16.0	38.3 ± 4.0	134.3 ± 22.8	260.3 ± 15.6
40	192.7 ± 15.8	33.7 ± 3.5	160.3 ± 17.2	268.3 ± 39.8
8	175.7 ± 22.0	33.7 ± 5.0	154.3 ± 29.1	297.3 ± 9.6
0	173.0 ± 22.6	32.0 ± 5.2	173.0 ± 10.8	274.3 ± 17.4
Positive control	1690.7 ± 53.4*^e^	1687.7 ± 46.7*^f^	1184.7 ± 52.0*^g^	1107.3 ± 81.0*^h^

No significant changes of revertants were observed at any concentrations of POM93 treatments in four tester strains. Statistical analysis: *P < 0.05 indicates a statistical difference with the control group by one-way ANOVA when the number of revertants was twice than control at TA97, TA98, TA100, and TA102

Positive control in w/o S9 condition: ^a^2,4,7-Trinitrofluorenone (TNF) 0.2 μg/plate; ^b^2,4,7-Trinitrofluorenone (TNF) 0.2 μg/plate; ^c^Methyl Methanesulfonate (MMS) 1.0 μL/plate; ^d^Methyl Methanesulfonate (MMS) 1.0 μL/plate; ^e^2-(2-furyl)-3-(5-nitro-2-fury)acrylamide (2-AF) 0.01 μg/plate; ^f^2-(2-furyl)-3-(5-nitro-2-fury)acrylamide (2-AF) 0.01 μg/plate; ^g^2-(2-furyl)-3-(5-nitro-2-fury)acrylamide (2-AF) 0.01 μg/plate; ^h^2-aminoanthracene (2AA) 10.0 μg/plate

Data summarized in Table 2 show that the single administration of 625, 1250, and 2500 mg/kg body weight dose of POM93 did not induce aberrant chromosomes of mice bone marrow after 24 h of exposure as compared to the control group. Compared to negative control and any of doses of POM93 treatments, a statistically significant (*P* < 0.05) increase in cyclophosphamide as a positive control in all types of aberrations (breaks, ring formation, gaps, fragmentations, and associations), while no increases or irregularities in chromosome aberrations were recorded in all treatment groups (Table 2).

**Table 2:** Effect of POM93 in chromosomal aberration in mice marrow cells

				Type of chromosome aberration				
	Groups	No. of cells	Aberration in %	Chro. frag.	Chro. break	Chro. gap	Chro. ring	Chro. Asso.
Positive control	Cyclophosphamide (40 mg/kg)	100	65.00 ± 2.07*	20	21	10	8	6
POM 93	2500 mg/kg	100	0.80 ± 1.03	1	3	1	2	1
	1250 mg/kg	100	0.30 ± 0.48	1	1	1	0	0
	625 mg/kg	100	0.60 ± 0.84	1	2	1	1	1
Negative control	Solvent double distilled water (DDW)	100	0.40 ± 0.70	1	2	0	1	0

The chromosomal aberrations were counted by 100 independent cells. For each treatment, at least 300 cells were examined as described in Section 2. The data are expressed as the Mean ± SD (*n* = 3). Significant difference between control treated group at *P < 0.05 versus control by one-way ANOVA

An increase in the frequency of micronucleated polychromatic erythrocytes (MNPCEs) was found in the positive control group compared with the negative control group (*P* < 0.05). However, there were no significant differences between the negative control and the groups treated with 625 g/kg b.w., 1250 g/kg b.w. and 2500 g/kg b.w. (*p* > 0.05) of POM93, respectively (Table 3). The ratio of PCEs to total erythrocytes (%) of the cyclophosphamide treatment was not significantly (*P* > 0.05) different from that of other groups. The micronucleus assay test revealed that POM93 did not induce numerical or structural chromosomal damage at the administered levels, with no acute toxicity being observed in the groups. These data indicated that neither mutagenicity nor clastogenicity was caused by POM93 treatments.

**Table 3:** Micronucleus assay of POM93 in vivo

Treatment	Dose (mg/kg b.w.)	Number of animals observed	Number of cells observed	Frequency of micronucleated polychromatic erythrocyte (‰)	Ratio of polychromatic erythrocyte (PCE) to total erythrocytes (%)
Positive control Cyclophosphamide	200	5	10,000	32.70 ± 3.16*	1.20 ± 0.14
POM93	625	5	10,000	5.00 ± 1.56	0.70 ± 0.31
	1250	5	10,000	3.50 ± 1.08	0.89 ± 0.27
	2500	5	10,000	5.70 ± 1.49	0.73 ± 0.22
Negative control	0	5	10,000	5.40 ± 1.51	0.55 ± 0.15

Both the ratios of polychromatic erythrocyte (PCE) to total erythrocytes (%) and the frequency of micronucleated polychromatic erythrocyte (‰) in treated group were not statistically different from those of the negative control animals, suggesting that the treatment with POM93 did not cause erythropoietic cell toxicity and genotoxicity in vivo. Data are expressed as mean ± S.D. (*n* = 6). Statistical analysis: *P < 0.05 indicate a statistical difference with the control group by one-way ANOVA

### No deaths or abnormalities were observed in the single-dose acute toxicity study

No deaths or hazardous signs of toxicity were recorded in the male and female rats during the 14-day period of observation after acute treatment by oral route with compound POM93. No abnormal changes in the ingestive behavior, body weight, and sensory nervous system responses were noted through the entire treatment. POM93 was found to be safe at doses of 2500 and 5000 mg/kg body weight, and therefore, the LD_50_ value was estimated to be greater than 5000 mg/kg when administered once orally to rats.

### POM93 was nontoxic but induced pharmacological effects in subchronic toxicity studies

Administration of POM93 at doses of 0, 62.2, 195.7, 587.0 mg/kg orally every 24 h for 90 days showed no treatment-related mortality or clinical signs of general toxicity. Regardless of treatment, no statistically significant differences were found in body weight gain (see Additional file 1: Table S1 in the SI), food consumption, or water intake (data not shown). Additional, there were no statistical changes in the external physical structure of the organs; their relative organ weight between the control group and the experimental groups was observed (see Additional file 1: Table S2 in the SI). The results of hematological parameters measured are presented in Table 4. All hematology measures of POM93-treated rats were not dramatically different from the controls. Serum biochemistry profiles are shown in Table 5; by the end of the testing period, most of the parameters were not affected by POM93 treatment. However, the activities of aspartate transaminase (AST) were increased at doses of 62.2 and 587.0 mg/kg in 13-week treated (*P* < 0.05). The levels of urea (UREA) and creatinine (CREA) were significantly higher in rats treated with 587.0 mg/kg POM93 than in the controls, as well as a high level of UREA was noted in rats treated with 195.7 mg/kg POM93. Furthermore, the activities of both alanine aminotransferase (ALT) and AST were increased in treated rats (at 62.2, 195.7, 587.0 mg/kg, respectively) after 2 weeks of drug discontinuation, and the levels of UREA and CREA were still statistically higher in rats treated with 587.0 mg/kg POM93 than in the controls.

**Table 4:** Effect of the subchronic 13-week oral administration of POM93 on hematological parameters in Wistar rats

Time (d)	Hematological parameters	POM93 (mg/kg)			
		Control water	62.2	195.7	587.0
90	WBC (× 10^9^/L)	14.20 ± 5.07	9.40 ± 2.91	12.20 ± 3.52	13.44 ± 4.33
	RBC (× 10^12^/L)	9.11 ± 0.77	8.28 ± 1.16	8.67 ± 0.73	8.79 ± 1.91
	PLT (× 10^9^/L)	657.40 ± 402.57	334.20 ± 296.94	356.40 ± 247.47	536.56 ± 338.81
	HB (g/L)	149.60 ± 11.88	142.50 ± 19.42	147.50 ± 15.36	144.67 ± 20.43
105	WBC (× 10^9^/L)	15.36 ± 3.45	15.52 ± 4.18	16.90 ± 12.35	24.20 ± 10.20
	RBC (× 10^12^/L)	8.96 ± 1.00	8.77 ± 0.91	8.72 ± 0.90	8.44 ± 0.81
	PLT (× 10^9^/L)	500.56 ± 185.23	490.90 ± 260.25	519.90 ± 343.95	587.67 ± 366.49
	HB (g/L)	149.56 ± 13.91	152.20 ± 12.93	143.90 ± 12.41	135.17 ± 16.38

Hematology profiles were within the normal range and unaffected by the POM93 treatment. Data are expressed as mean ± S.D. (*n* = 20)

**Table 5:** Effect of the subchronic 13-week oral administration of POM93 on biochemical parameters in Wistar rats

Time (d)	Biochemical parameters	POM93 (mg/kg)			
		Control water	62.2	195.7	587.0
90	ALT (U/L)	108.40 ± 13.78	121.30 ± 14.85	112.40 ± 14.26	119.90 ± 10.83
	AST (U/L)	154.50 ± 20.66	192.90 ± 22.24*	169.40 ± 28.56	197.60 ± 46.37*
	ALP (U/L)	136.60 ± 12.90	129.80 ± 48.60	106.70 ± 24.87	121.10 ± 29.89
	T-B IL (mmol/L)	0.32 ± 0.15	0.34 ± 0.22	0.44 ± 0.29	0.51 ± 0.25
	D-B IL (mmol/L)	0.68 ± 0.30	0.88 ± 0.56	0.85 ± 0.51	0.74 ± 0.45
	TP (mmol/L)	75.80 ± 3.92	78.55 ± 5.90	80.55 ± 7.64	82.10 ± 8.14
	UREA (mmol/L)	7.90 ± 1.65	8.81 ± 1.08	9.97 ± 2.19*	10.23 ± 0.65
	CREA (mmol/L)	54.20 ± 8.09	52.80 ± 8.69	56.80 ± 7.34	79.70 ± 13.32^*△^
	CHOL (mmol/L)	1.64 ± 0.15	1.53 ± 0.33	1.62 ± 0.40	1.67 ± 0.33
	TG (mmol/L)	0.98 ± 0.11	1.07 ± 0.17	0.90 ± 0.13	1.10 ± 0.25
	GLU (mmol/L)	1.87 ± 0.13	1.89 ± 0.29	1.70 ± 0.35	1.78 ± 0.30
105	ALT (U/L)	132.90 ± 20.10	158.60 ± 17.18*	159.90 ± 19.11*	162.30 ± 8.16*
	AST (U/L)	165.40 ± 15.05	200.20 ± 21.69*	200.00 ± 16.95*	242.30 ± 32.88^*△^
	ALP (U/L)	141.60 ± 23.16	195.80 ± 63.17	197.40 ± 83.28	240.0 ± 165.90
	T-B IL (mmol/L)	0.51 ± 0.30	0.39 ± 0.33	0.52 ± 0.28	0.75 ± 0.43
	D-B IL (mmol/L)	0.76 ± 0.21	1.17 ± 0.76	0.93 ± 0.38	0.85 ± 0.49
	TP (mmol/L)	76.57 ± 6.51	80.51 ± 3.44	79.41 ± 4.55	80.03 ± 9.74
	UREA (mmol/L)	7.32 ± 1.32	8.00 ± 1.80	8.26 ± 2.04	10.63 ± 1.14^*△^
	CREA (mmol/L)	40.10 ± 3.25	48.80 ± 6.97*	51.30 ± 7.54*	62.60 ± 3.77^*△^
	CHOL (mmol/L)	1.35 ± 0.19	1.52 ± 0.32	1.61 ± 0.24	1.49 ± 0.60
	TG (mmol/L)	1.13 ± 0.26	0.85 ± 0.30	1.04 ± 0.33	1.03 ± 0.46
	GLU (mmol/L)	4.26 ± 0.84	4.21 ± 0.65	3.91 ± 0.51	4.48 ± 0.73

Most serum biochemical parameters were within the normal range, except the pharmacological effects ALT in 90 d treated groups, AST, UREA, and CREA in 90 and 105 days treated groups, after a 13-week POM93 oral treatment. The results are shown as average ± S.D. (*n* = 20), *P < 0.05 versus control and ^△^ *P* < 0.05 versus 62.2 mg/kg groups by one-way ANOVA

### Microscopic evaluation of tissue sections did not show abnormal histopathology changes

**Fig. 1:**
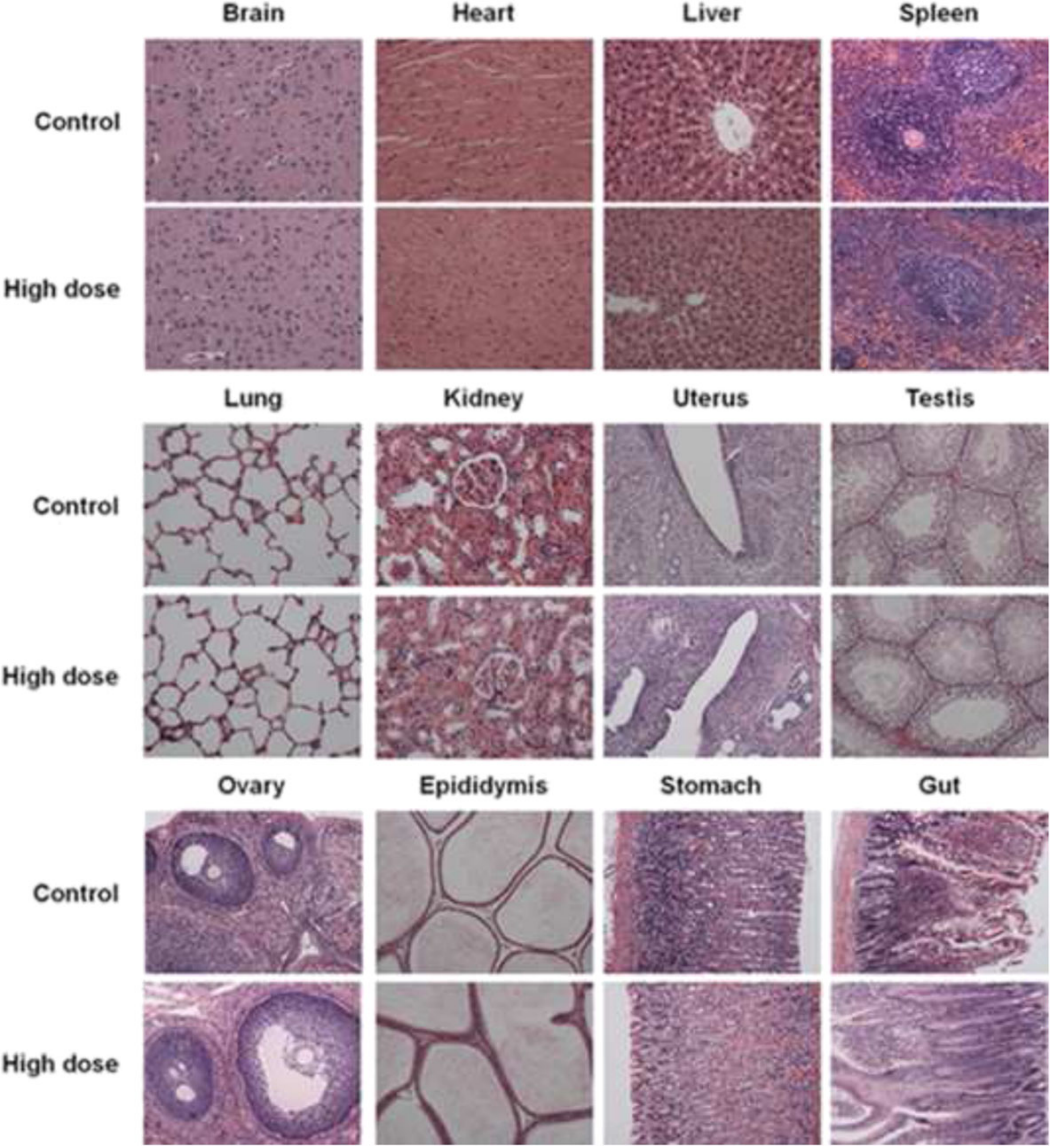
Histopathological sections of major organs in the 90 day repeated dose toxicity assessment. There were no histopathological findings, which distinguished the POM93-treated animals from the controls. Magnification, × 100

Histopathological sections of brain heart, liver, spleen, lungs, kidneys, uterus (or testis), ovary (or epididymis) stomach, and gut are shown in Fig. 1. No lesions, inflammation, or pathological changes related to treatment with POM93 were observed in the organs of the rats from the POM93-treated groups compared with the untreated group. In general, there was no evidence of abnormal features both in the various organs and the structures around it; similar results were obtained from the analysis of the experimental groups and control group.

## Discussion

The priority in research on POMs as potential drugs for the treatment of various diseases is assessment of the safety of POM compounds. Thus, systematic toxicological analysis must be performed using different experimental models to predict the toxicity of a POM-based compound and develop a criterion set for selecting a safe dose in humans. The toxic effect of drugs in animals and humans are associated with several serious detrimental effects, including hematological effects, renal effects, reproductive problems, gastrointestinal disturbances, and mutagenic effects caused by different dose levels. Therefore, for the determination of the overall toxicity of POM compound, investigating adverse effects of a single and repeated dose of POM in experimental animals and the mutagenicity in mutant strains of bacteria may be more relevant. In certain situations, few scientific evidence has been established to evaluate the safety of POM; in our previous study, a potential compound with broad spectrum antiviral effective on anti-HBV, −HCV,-HSV [6, 21] and anti-HIV named POM93 has been considered non-toxic to the cells in vitro, and the mechanism of its anti-HBV activity was clarified through the X-ray nanotomography experiments [23]. However, cytotoxicity experiments in vitro cannot generate all the toxicity data, and we now provide data on the acute and chronic toxicity of POM93. The present study was designed to examine the effect of oral acute and subchronic toxicity of POM93 and its genotoxicity in rodents and bacterial mutant strains.

As an initial step, a LD_50_ test was conducted during evaluation of the toxic effect of POM93, measured by oral single-dose administration. In the current study, oral treatment with POM93 was well tolerated. A dose of 5000 mg/kg POM93 administered to rats did not cause any acute toxicities; changes in host mortality and any abnormal behavior were not observed for up to 14 days. These results revealed that the LD_50_ value of POM93 was estimated to be greater than 5000 mg/kg. According to the labelling and classification of acute systemic toxicity recommended by the OECD, POM93 was assigned class 5 status (LD_50_ > 5000 mg/kg), i.e., in the lowest toxicity class [33]. Because a substance with an LD_50_ of 5000 mg/kg or above is generally considered to be nontoxic, further investigation was conducted to evaluate the subchronic toxicity of POM93 for up to 13 weeks in rats to prepare the comprehensive toxicology data of this potential bioactive compound.

The results of the subchronic 90-day oral toxicity study of POM93 demonstrated no adverse clinical signs or negative influence on the behavior and mortality in the treatment groups. Comparison of food consumption, water intake and body weight gain in rats revealed no statistically significant alteration between POM93-treated rats and controls. Because body weight changes serve as a sensitive index for the general health status of animals [34], the result suggested that POM93 did not alter food intake through appetite suppression. In addition, the relative organ weights were examined in this study, and the results revealed that the weights of the organs did not significantly differ from those of the control group, indicating that there was no interference on the relative organ weights of the treated animals at any of the doses tested. Furthermore, 2 weeks of drug discontinuation, there was no reduction in the body weights and relative organ weights as well as after 90 days of exposure. Based on these results, we therefore conclude that POM93 is safe even with the highest studied dose (587.0 mg/kg), and it was non-toxic to all possible target organs during the entire period of experiment.

The serum hematology and clinical biochemistry analyses were performed to evaluate the possible alterations in liver and kidney functions induced by POM93. Our results showed no significant differences (*P* < 0.05) in hematological parameters among rats treated with POM93 at doses of 0, 62.2, 195.7, and 587.0 mg/kg orally every 24 h for 90 days; this indicates that there was no adverse effect induced by POM93, such as cell damage and inflammation. Almost all biochemical parameters analyzed in the present research showed normal values with respect to the untreated group. However, the activities of AST were increased at doses of 62.2, and 587.0 mg/kg (*P* < 0.05) compared with the controls for 90 consecutive days. The change in AST activity was not dose dependent because they were only observed in the group treated with low dose (62.2 mg/kg b.w.) and high dose (587.0 mg/kg b.w.), but not in the group treated with the medium dose (195.7 mg/kg b.w.). Serum ALT and AST activities are routinely used as clinical endpoints indicative of hepatotoxicity [35, 36]. Mild forms of toxic hepatitis may not cause any symptoms or pathologic changes and may be detected only by blood tests. Generally, clinically significant liver injury is often defined as ALT > 3 times the upper limit of normal (ULN) [37]. However, in the present study, the ALT level (162.3 U/L) at the highest dose didn’t reach up to the diagnostic criteria of liver damage, compared to the control group (132.9 U/L). Histological samples obtained from rats with biochemical abnormalities in the POM93 treatment group had not shown any tissue and cellular damage, which suggest that the slightly elevated serum AST in our test probably fall short of being indicative of liver injury.

The increased levels of UREA and CREA in the rats treated with 587.0 mg/kg POM93 may be because of mild renal damage at the highest dose, and the finding should be further clarified by histopathological examination. Notably, after 2 weeks of drug discontinuation (the 105th day), we found the activities of both ALT and AST were increased in treated rats (at 62.2, 195.7, 587.0 mg/kg); these results indicate that POM93 may have latent effect on the rats’ liver. Moreover, we confirmed these results by histopathological examination of excised tissues [brain, heart, liver, spleen, lungs, kidneys, uterus (or testis), ovary (or epididymis), stomach, and gut] harvested from POM93-treated and control animals. Normal organ architecture was observed in all the vital organs by light microscope using multiple magnification powers, and the results revealed that none of the organs from the POM93-treated rats showed any alteration in cell structure, inflammation, or any unfavorable effects on subchronic toxicity study. Remarkably, the POM93-treated rats had higher levels of serum AST, UREA, and CREA, but through analysis of histopathological examination results, no significant damage was induced by treatment with POM93 to the liver and kidney parenchymal cells. Therefore, this strongly suggests that there are no obvious detrimental effects or morphological disturbances caused by daily oral administration of POM93 for 90 days, even at the highest tested dose of 587.0 mg/kg.

In the current study, mutagenicity (Ames test in four bacterial mutant strains) and clastogenicity (mice bone marrow cellular chromosome aberration and polychromatic erythrocyte micronucleus test in vivo) were determined in accordance with international guidelines. Neither mutagenic nor clastogenic effects were found (at concentrations up to 5000 μg/plate) or in vivo (at doses up to 2500 mg/kg b.w.), suggesting that POMs, especially POM93 is not genotoxic.

## Conclusion

In light of experimental findings, we may conclude that POM93 orally administered to Wistar rats was safe at the various doses and that no POM93-related severe toxicity was detected even at the highest dose investigated in both acute (5000.0 mg/kg) and subchronic (587.0 mg/kg) levels. In summary, POM93 did not cause mortality or significant visible signs of toxicity on the respiratory system, digestive system, reproductive system, and other physiological functions following 90 days of gavage administration in rats. Furthermore, POM93 did not induce genotoxic injury or mutagenic effects even at extremely high concentrations in vitro and in vivo. Cumulatively, these data suggest that the consumption of POM93 poses a low risk of potential health risks in humans. Thus, the development of novel POM-based drugs has great prospects in the future.

## Additional files

Additional file 1: Supporting information for POM93 characterization (**Figures S1-S2**) and subchronic toxicity (**Table S1-S2**). (DOCX 54 kb)

## Abbreviations

ALT: Alanine aminotransferase
ANOVA: Analysis of variance
anti-HBV: Anti-hepatitis B virus
anti-HCV: Anti-hepatitis C virus
AST: Alanine aminotransferase
CREA: Creatinine
H & E: Hematoxylin and eosin
LD_50_: The median acute toxicity values
OECD: Organization for Economic Cooperation and Development
POMs: Polyoxometalates
SD: Standard deviation
UREA: Urea
