# Supplementary material for: Genotoxicity and acute and subchronic toxicity studies of a bioactive polyoxometalate in Wistar rats
Source: BMC Pharmacol Toxicol. 2017 Apr 5;18:26. doi: 10.1186/s40360-017-0133-x (PMC5382445; doi:10.1186/s40360-017-0133-x)
Supplement: Supplementary file 1 — Supporting information for POM93 characterization (Figures S1-S2) and subchronic toxicity (Table S1-S2). (DOCX 54 kb) [file 40360_2017_133_MOESM1_ESM.docx]

**Genotoxicity, Acute and Subchronic Toxicity Studies of a Bioactive Polyoxometalate in Wistar Rats**

(Supporting information)

Xiaofeng [Qu](http://www.ncbi.nlm.nih.gov/pubmed/?term=Qu%20X%5BAuthor%5D&cauthor=true&cauthor_uid=24921932), Kun [Xu](http://www.ncbi.nlm.nih.gov/pubmed/?term=Xu%20K%5BAuthor%5D&cauthor=true&cauthor_uid=24921932), Chao Zhao, Xiuling [Song](http://www.ncbi.nlm.nih.gov/pubmed/?term=Song%20X%5BAuthor%5D&cauthor=true&cauthor_uid=24921932), Jinhua [Li](http://www.ncbi.nlm.nih.gov/pubmed/?term=Li%20J%5BAuthor%5D&cauthor=true&cauthor_uid=24921932), Li Li，Wei Nie, Hao Bao, Juan Wang,^*^ Fenglan Niu and Juan Li

School of Public Health, Jilin University, Changchun, Jilin, China

* E-mail: [jwang0723@jlu.edu.cn](mailto:jwang0723@jlu.edu.cn) (JW)

**Figure S1.** XRD spectrum of POM93

FTIR (KBr): 3429, 2357, 1623, 990, 958, 868, 789, 673, 595, 534, 474 cm-1.

**Figure S2.** FTIR spectrum of POM93

Table S1 Body weight changes for rats in subchronic 13-week oral toxicity test of POM93

| Dose  (mg/kg) | No. of Animals | Body weight (g) | | | |
| --- | --- | --- | --- | --- | --- |
|  |  | 0 d | 45 d | 90 d | 105 d |
| 0 | 20 | 203.07±17.54 | 310.10±45.46 | 355.85±59.40 | 371.90±66.32 |
| 62.2 | 20 | 205.27±13.44 | 299.73±42.28 | 344.95±50.63 | 375.30±64.03 |
| 195.7 | 20 | 211.47±18.90 | 297.63±40.02 | 364.90±63.87 | 384.20±69.97 |
| 587.0 | 20 | 212.27±13.47 | 299.13±42.15 | 347.85±51.54 | 373.30±68.90 |

Table S2 Effects of the subchronic 13-week oral administration of POM93 on organ weights in Wistar rats

| Time (d) | Organ | Organ weight (g) | | | | | | | |
| --- | --- | --- | --- | --- | --- | --- | --- | --- | --- |
|  |  | Control water | | POM93 (mg/kg) | | | | | |
|  |  |  |  | 62.2 | | 195.7 | | 587.0 | |
| 45 | Heart | 0.37±0.05 | | 0.38±0.05 | | 0.38±0.06 | | 0.36±0.07 | |
|  | Liver | 3.35±0.44 | | 3.35±0.33 | | 3.33±0.30 | | 3.01±0.55 | |
|  | Spleen | 0.48±0.18 | | 0.47±0.11 | | 0.42±0.07 | | 0.49±0.17 | |
|  | Lung | 0.60±0.10 | | 0.56±0.15 | | 0.56±0.10 | | 0.59±0.25 | |
|  | Kidney | 0.66±0.06 | | 0.67±0.09 | | 0.69±0.10 | | 0.66±0.09 | |
|  | Uterus | 0.19±0.05 | | 0.15±0.04 | | 0.15±0.04 | | 0.18±0.03 | |
|  | Testis | 0.97±0.10 | | 0.96±0.18 | | 1.10±0.20 | | 1.00±0.14 | |
|  | Ovary | 0.05±0.01 | | 0.04±0.01 | | 0.04±0.01 | | 0.05±0.01 | |
|  | Epididymis | 0.34±0.04 | | 0.31±0.06 | | 0.32±0.05 | | 0.28±0.04 | |
| 90 | Heart | 0.41±0.07 | | 0.36±0.05 | | 0.65±0.05 | | 0.39±0.05 | |
|  | Liver | 2.73±0.50 | | 2.87±0.45 | | 2.57±0.21 | | 2.68±0.21 | |
|  | Spleen | 0.37±0.07 | | 0.40±0.16 | | 0.37±0.08 | | 0.35±0.06 | |
|  | Lung | 0.49±0.10 | | 0.49±0.11 | | 0.49±0.06 | | 0.50±0.05 | |
|  | Kidney | 0.57±0.07 | | 0.58±0.09 | | 0.57±0.05 | | 0.64±0.06 | |
|  | Uterus | 0.07±0.08 | | 0.11±0.13 | | 0.09±0.10 | | 0.08±0.09 | |
|  | Testis | 0.39±0.45 | | 0.47±0.50 | | 0.39±0.45 | | 0.48±0.52 | |
|  | Ovary | 0.02±0.02 | | 0.02±0.02 | | 0.02±0.03 | | 0.02±0.03 | |
|  | Epididymis | | 0.14±0.16 | | 0.18±0.19 | | 0.13±0.14 | | 0.14±0.14 |
| 105 | Heart | 0.42±0.06 | | 0.40±0.09 | | 0.39±0.05 | | 0.37±0.07 | |
|  | Liver | 3.01±0.37 | | 3.08±0.86 | | 2.68±0.36 | | 3.16±0.83 | |
|  | Spleen | 0.32±0.06 | | 0.41±0.20 | | 0.38±0.10 | | 0.38±0.12 | |
|  | Lung | 0.50±0.06 | | 0.49±0.10 | | 0.49±0.10 | | 0.48±0.07 | |
|  | Kidney | 0.57±0.07 | | 0.55±0.07 | | 0.53±0.06 | | 0.55±0.06 | |
|  | Uterus | 0.12±0.13 | | 0.13±0.14 | | 0.13±0.18 | | 0.15±0.13 | |
|  | Testis | 0.46±0.49 | | 0.39±0.43 | | 0.42±0.45 | | 0.49±0.54 | |
|  | Ovary | 0.02±0.02 | | 0.02±0.02 | | 0.02±0.03 | | 0.03±0.02 | |
|  | Epididymis | | 0.16±0.17 | | 0.14±0.15 | | 0.14±0.15 | | 0.14±0.15 |
